# Evaluation of microleakage of mineral trioxide aggregate and biodentine as apical barriers in simulated young permanent teeth

**DOI:** 10.1186/s12903-024-04817-2

**Published:** 2024-09-16

**Authors:** Medha Roy, Sandeep A. Bailwad, Akash Bhatnagar, Sushma Singh, Ali A. Assiry, Roshan Noor Mohamed, Sakeenabi Basha, Niher Tabassum Snigdha, Mohmed Isaqali Karobari

**Affiliations:** 1 Department of Pediatric and Preventive Dentistry, Teerthanker Mahaveer Dental College and Research Centre, Moradabad, Uttar Pradesh India; 2 Preventive Dental Science Department, Faculty of Dentistry, Najran University, Najran, 55461 Saudi Arabia; 3 Department of Pediatric Dentistry, Faculty of Dentistry, Taif University, Taif, Saudi Arabia; 4 Preventive Dentistry Department (Community Dentistry Division), Faculty of Dentistry, Taif University, Taif, Saudi Arabia; 5 Department of Dental Research, Saveetha Medical College and Hospital, Saveetha Institute of Medical and Technical Sciences, Chennai, Tamil Nadu 602105 India; 6 Department of Conservative Dentistry & Endodontics, Saveetha Dental College & Hospitals, Saveetha Institute of Medical and Technical Sciences, Saveetha University, Chennai, 600077 India; 7 Department of Restorative Dentistry & Endodontics, Faculty of Dentistry, University of Puthisastra, Phnom Penh, 2211 Cambodia

**Keywords:** Open apices, Microleakage, MTA, Biodentine

## Abstract

**Background:**

Apexification is a procedure that promotes apical closure by forming mineralized tissue in the apex region of a nonvital young permanent tooth. Calcium silicate-based cement like Mineral trioxide aggregate (MTA) and Biodentine are commonly employed as apical barriers to facilitate this process. Microleakage, defined as the leakage along the junction between the canal wall and filling material, is a crucial aspect to assess in MTA and Biodentine applications as apical barriers, as it directly impacts the prevention of bacterial seepage and maintenance of structural integrity. The current study aims to assess the microleakage of MTA and Biodentine when used as apical barriers in simulated young permanent teeth.

**Methods:**

From a total of 128 extracted teeth, 114 were selected for the study and randomly allocated into three groups: G1 (MTA), G2 (Biodentine), and G3 (Control), with 38 teeth per group. After excluding 5 teeth from each group due to issues such as canal calcification, breakage during sectioning, and procedural errors, 33 teeth were analyzed to ensure equal distribution. To simulate young permanent teeth, samples were instrumented using a person-reamer with a diameter of 1.7 mm. A 4 mm thick apical plug of MTA and Biodentine was placed in G1 and G2, respectively, while G3 was the control group. Apical microleakage in all experimental groups was assessed using a dye penetration method. Specimens were longitudinally sectioned and examined under a stereomicroscope with graded eyepiece.

**Results:**

The Kruskal-Wallis test revealed variations in mean apical microleakage among the groups: G1 recorded 0.67, G2–0.16, and G3–1.62, with G2 showing the lowest value and G3 group exhibiting the highest.

**Conclusions:**

Biodentine was found to excel in its ability to create a secure seal and function effectively as an apical barrier in simulated young permanent teeth. These results underscore its potential as a highly efficient material for dental applications, particularly in scenarios requiring reliable sealing and barrier formation in the root canal system of developing permanent teeth.

## Introduction

After a permanent tooth erupts, it undergoes a developmental process that spans approximately three years to achieve full root development, which includes the crucial step of apex closure. Various factors can significantly affect the health of the tooth pulp, with trauma and dental caries emerging as primary contributors to adverse outcomes in young permanent teeth [1].

Apexification, inducing apical closure, is one method of treating necrotic young permanent teeth to establish conditions more conducive to traditional root canal filling [2]. Due to the multiple drawbacks of Calcium hydroxide apexification, single-step apexification became popularised. An artificial apical barrier is used in a single-step apexification alternative to calcium hydroxide apexification.

Mineral trioxide aggregate (MTA) has been the subject of intensive research over the last decade, and it has been suggested as a potential solution to numerous clinical endodontic difficulties. However, it also has several disadvantages: longer setting time, inflated cost & lack of good handling characteristics [3]. The more recent calcium-silicate-based material is Biodentine, which has the same composition as MTA and is currently used as an apical barrier. Biodentine possesses notable clinical characteristics such as improved sealing ability, greater compressive strength, less porosity, more significant density, bioactivity, and fast generation of calcium hydroxide [4, 5].

Materials, tools, and methods innovations keep endodontic treatment procedures sophisticated, increasing the likelihood of predictable clinical success. However, despite these developments, clinical shortcomings/failures continue to exist [6]. Microleakage is the most frequent cause of endodontic failure, defined as the clinically undetectable passage of bacteria, fluids, molecules or ions between the tooth and the restorative or filling material [7]. Methylene blue dye penetration is one of the most frequently employed methods for measuring microleakage. Since the dye molecules in the methylene blue solution are 10^3^ times smaller than those in bacteria, 144.5% of the test specimens may demonstrate excessive dye penetration [8].

The assessment of microleakage in MTA and Biodentine as apical barriers is necessary to overcome the seepage of bacterial load and maintain integrity. Nepal et al. found no statistically significant difference in the mean microleakage between MTA and Biodentine. Thapaliya et al. demonstrated that Biodentine exhibited superior sealing ability in their study. Rafaei et al. concluded that Biodentine showed superior sealing efficiency compared to other materials in their research. Conversely, Ozbay et al. reported lower levels of microleakage associated with MTA compared to Biodentine. These findings highlight varying perspectives on the sealing capabilities of MTA and Biodentine in endodontic applications, suggesting the need for further comparative studies to clarify their respective advantages and limitations [9–12]. Thus, considering the importance of the sealing ability of both MTA and Biodentine and decreasing the microleakage in young permanent teeth is clear. In order to enhance sealing efficiency, this study evaluated the microleakage performance of MTA and Biodentine as apical barriers. The research focused on simulated young permanent teeth, aiming to assess and compare how effectively these materials prevent the passage of fluids and microorganisms along the root canal. A null hypothesis states that no statistical significance exists in the microleakage between MTA and Biodentine when used as apical barriers in simulated young permanent teeth.

## Methods

The current research was performed in the Department of Pedodontics and Preventive Dentistry, Teerthanker Mahaveer Dental College & Research Centre affiliated with TMU, Moradabad. The ethical committee of TMDC&RC, Moradabad, reviewed the study & gave its approvalvide REF. NO: TMDCRC/IEC/21–22/PDD2. Following a rigorous power analysis, the study’s sample size was determined using G*Power software, version 3.1.9.6 (developed by Franz Faul, University of Kiel, Germany). It was calculated that all the subjects would be necessary to achieve a statistical power of 95% for detecting significant differences. This calculation was based on an effect size of 0.40 and a type I error rate of 5%, ensuring robustness in the study’s ability to draw reliable conclusions from the data collected.

The inclusion criteria for the study were sound-extracted maxillary anterior teeth and *s*ingle canal teeth with a minimum of 10–12 mm root lengths. Exclusion criteria were cracks and defects, carious teeth, teeth with resorption, teeth with calcified canals, and teeth with developmental malformation.

**Fig. 1:**
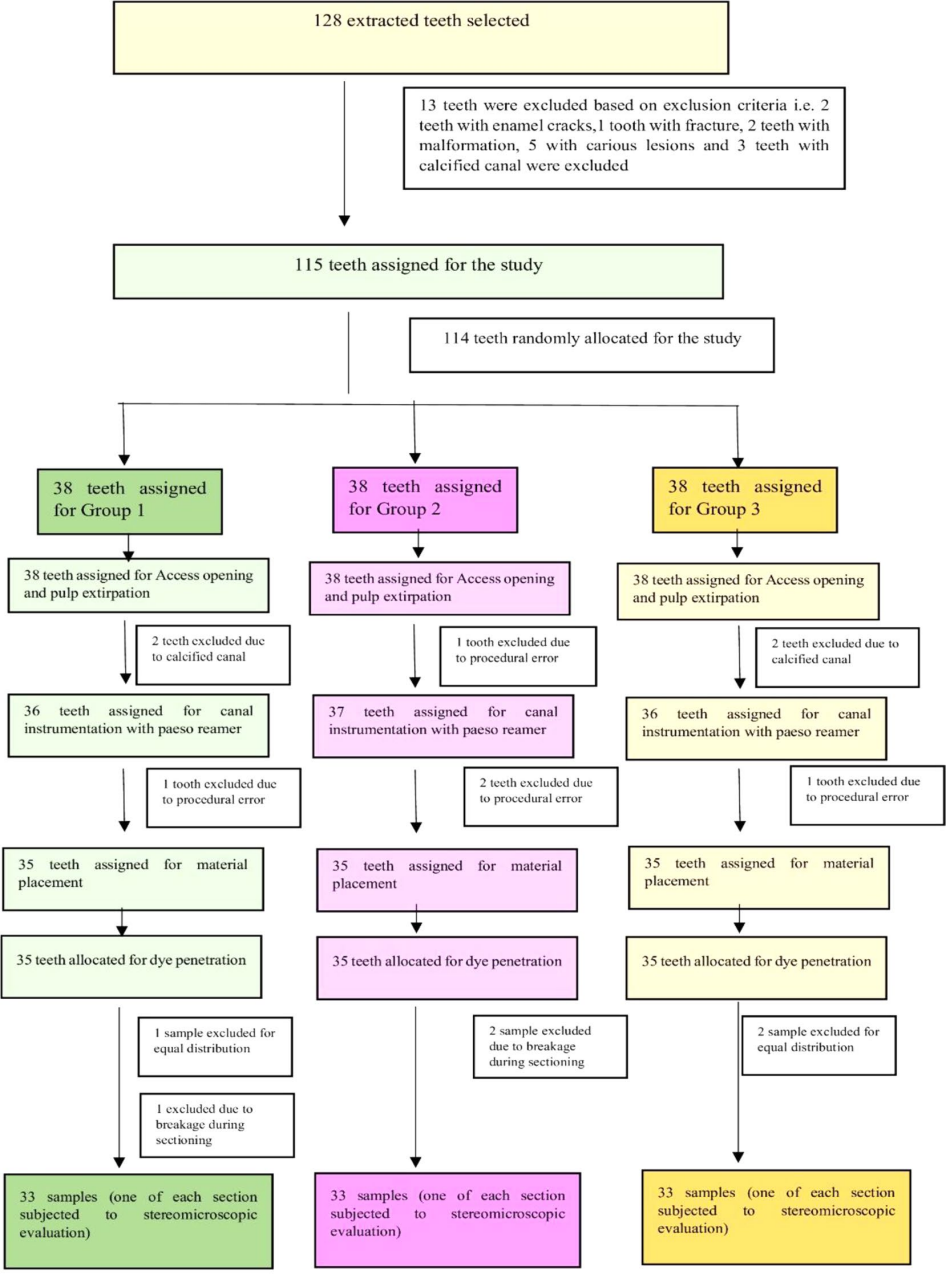
Flow chart (Checklist for Reporting In-vitro Studies)

Right after extraction, all teeth underwent a meticulous cleaning process involving brushing under running tap water. Surface debridement using a hand scaler, followed by an ultrasonic scaler and rubber cup with applied slurry pumice. Subsequently, the specimens were immersed in a 3% sodium hypochlorite solution. After that, the specimens were kept in a normal saline solution for 7days. Specimens were selected in the study according to the inclusion criteria. The allocation of collected tooth specimens for the following study was depicted in a Flow chart (Fig. 1). A total of 128 extracted teeth were collected for the study. Based on the inclusion and exclusion criteria, 115 teeth were selected. One tooth was excluded to ensure equal distribution among the three groups. Thus, 114 teeth were randomly allocated into three groups, with 38 teeth in each group, following the CRIS guidelines (Checklist for Reporting In-vitro Studies).

- Group 1: Five teeth were excluded (2 due to calcified canals, 1 due to procedural error, 1 due to breakage during sectioning, and 1 for equal distribution).
- Group 2: Five teeth were excluded (3 due to procedural errors and 2 due to breakage during sectioning).
- Group 3: Five teeth were excluded (2 due to calcified canals, 1 due to procedural error, and 2 for equal distribution).

Finally, based on the designated treatment protocols, 33 teeth were analyzed in each group for the study denoted as G1, G2, and G3.

**G1** – open apices of the prepared canal were treated with MTA and obturated (33teeth).

**G2** – open apices of the prepared canal were treated with Biodentine and obturated (33teeth).

**G3** –open apices of the prepared canal were obturated with gutta-percha only (33teeth).

**Fig. 2:**
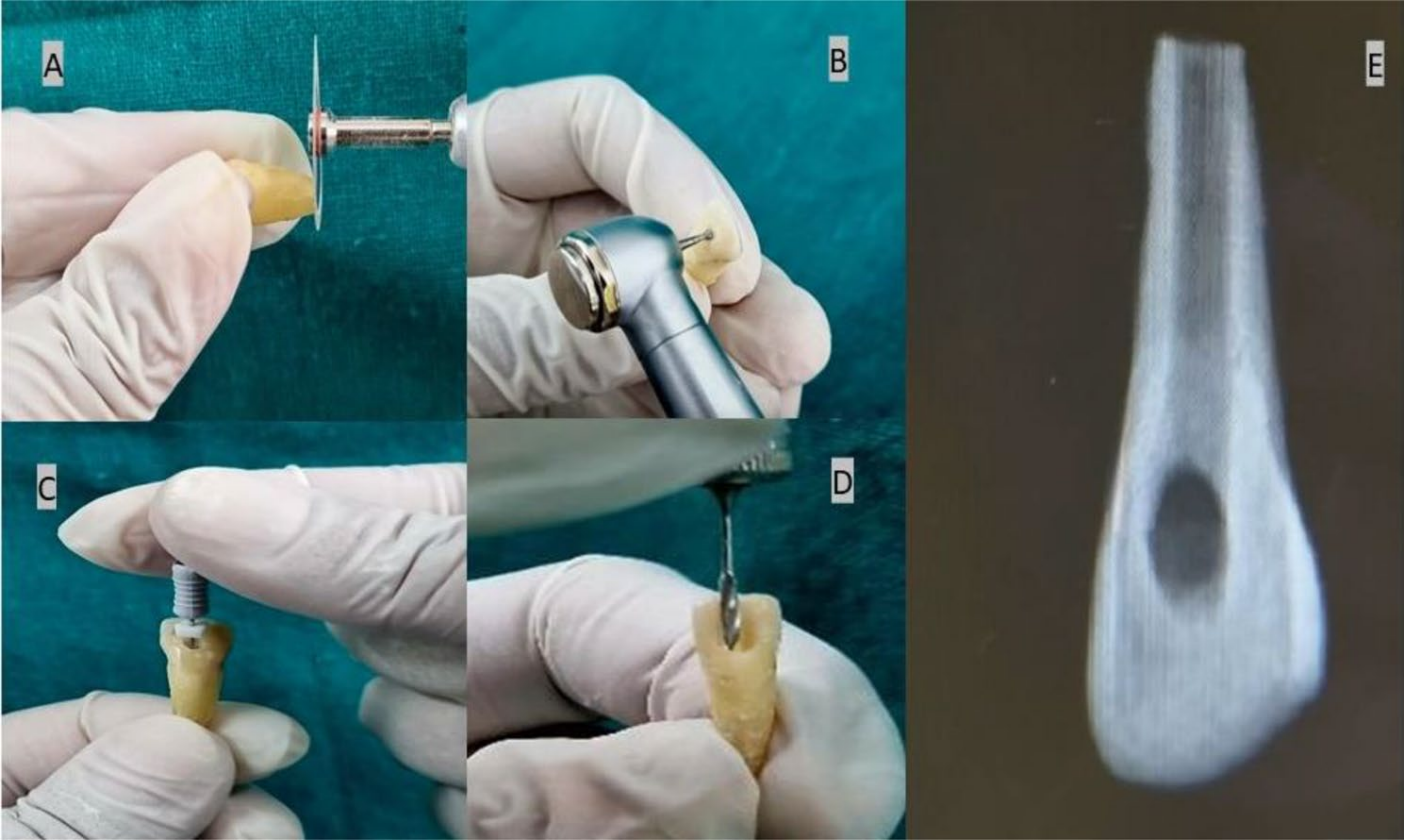
**A**. Root apices were removed using diamond disk bur, **B**. Access opening was done using round bur, **C**. Canals were navigated using 15 K file, **D**. Canals were enlarged with Peeso Reamer of diameter 1.7 mm, E. Simulated to young permanent tooth

The root apices of each group were excised using a 2 mm diamond disk bur(Kerr, USA), starting from the apices to simulate an open apex. Subsequently, the access opening was created with a round bur, and deroofing was performed using an Endo Z bur (Dentsply, Switzerland). Following that, canals were navigated using a 15k file (Dentsply, Switzerland). Subsequently, the pulp was extirpated using a barbed broach. To mimic immature permanent teeth, the root canals were prepared using a peeso reamer (Mani. Inc, New Delhi) in an orthograde manner (sizes 1 to 6) followed by a retrograde approach (sizes 1 to 6). This method aimed to simulate an open apex similar to Cvek’s stage 3 root development, achieving a diameter of approximately 1.7 mm, using water coolant during instrumentation. The canals were irrigated with 3% sodium hypochlorite throughout the preparation process. (PrevestDenPro, India). For smear layer removal, 17% EDTA (WaldentRCTprep, India) was applied to the canals for 1 min. Subsequently, the canals were again irrigated using 3% sodium hypochlorite. The canals were finally irrigated with normal saline and dried with paper points. Following this, all the teeth were stored for 1 week at 37 °C and 100% humidity (Fig. 2).

**Fig. 3:**
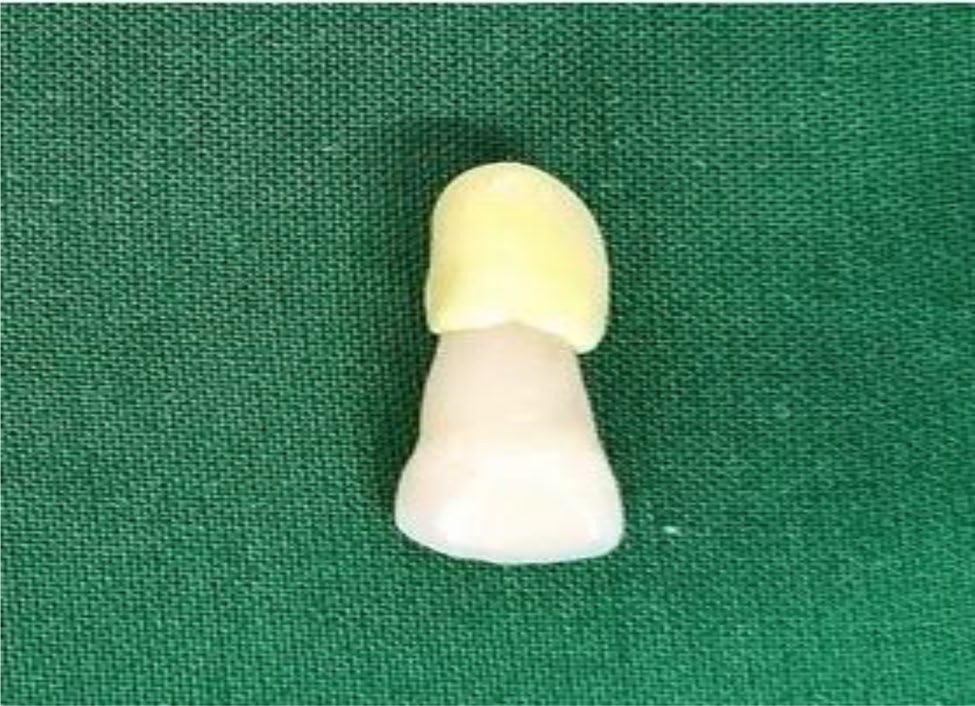
Putty was used simulate periapical region

**Fig. 4:**
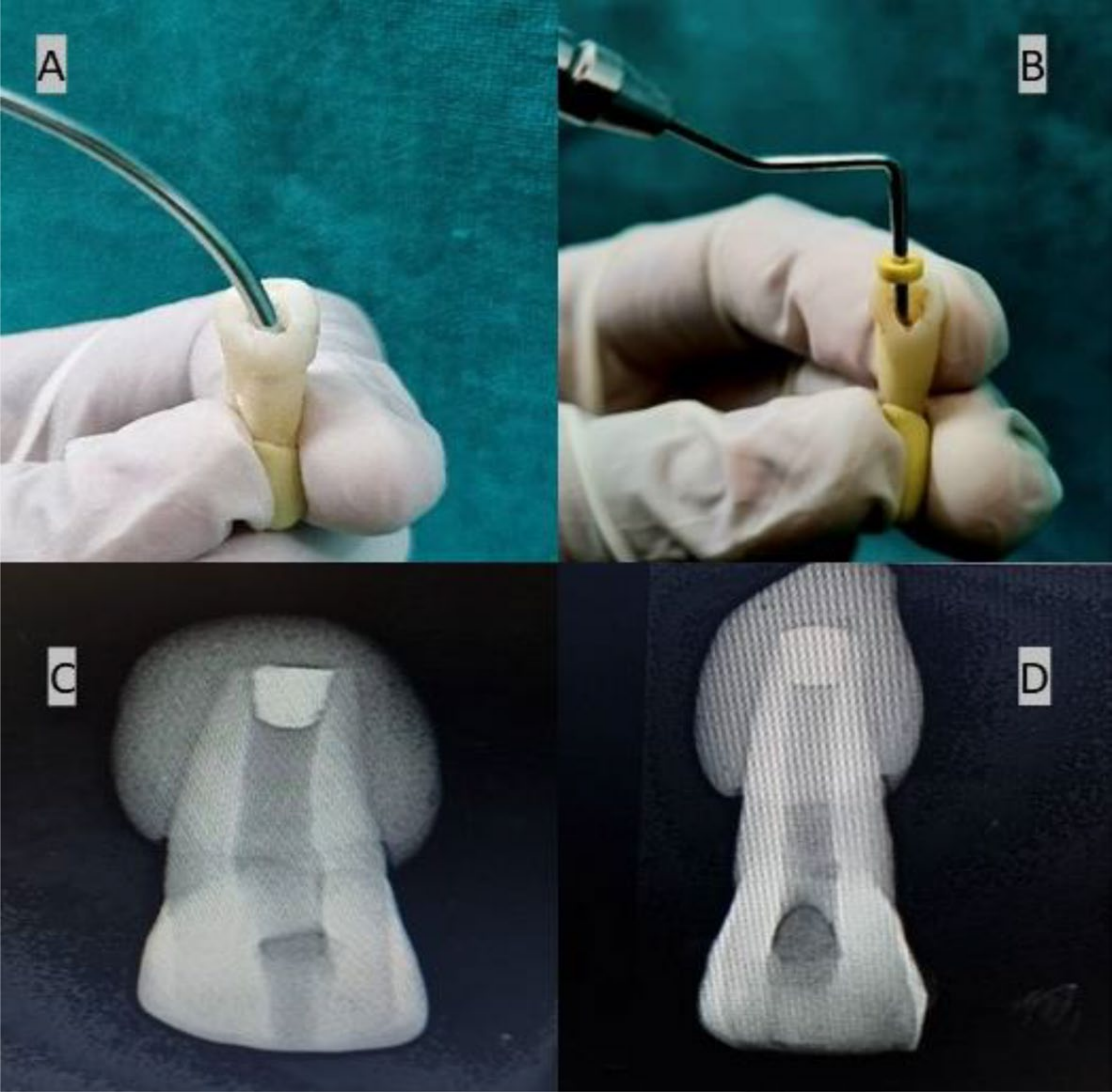
**A**. MTA carrier was used to place the materials in to the canal, **B**. Hand plugger was used to condense the materials, **C**&**D**. 4 mm thick apical plug in G1, G2 respectively

**Fig. 5:**
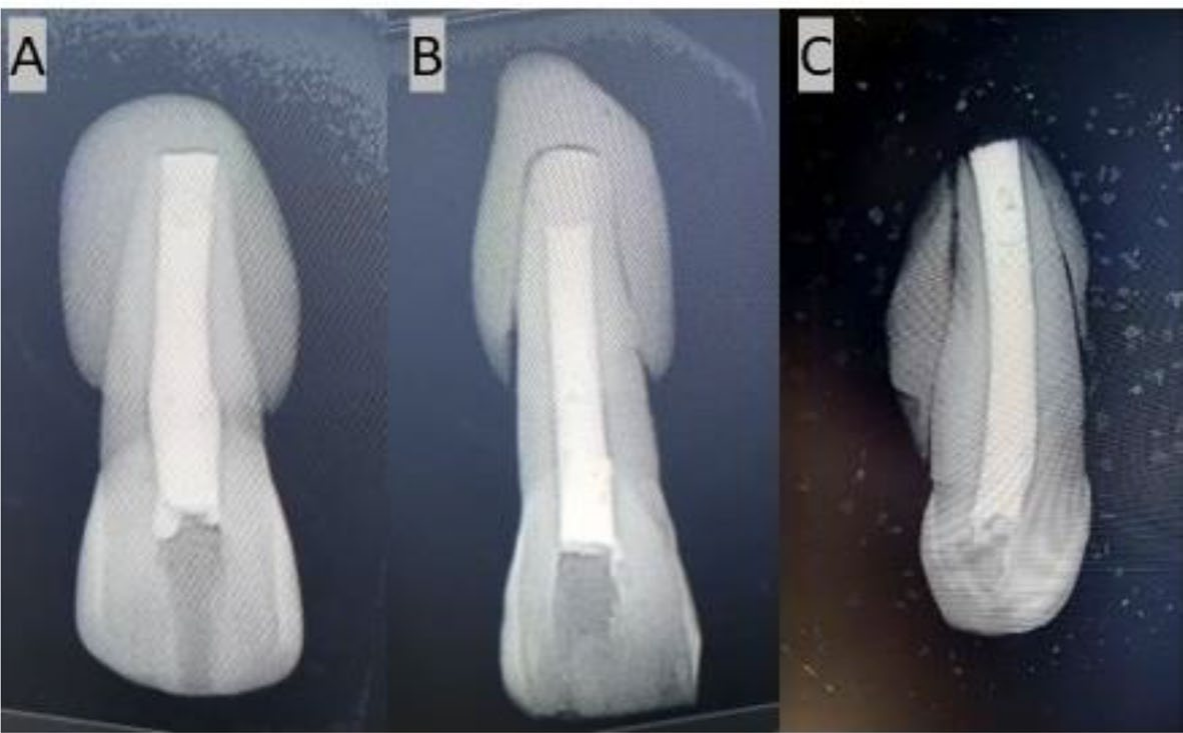
(**A**-**C**) Thermoplasticized gutta percha was used to obturate the canals G1, G2, G3 respectively

Putty was used in each group to simulate the periapical region (Fig. 3).MTA(Kids - e - Dental, Santacruz West, Mumbai, Maharashtra) and Biodentine(Septodont, Saint-Maur-des-Fossés, France) were mixed according to the manufacturer’s recommendation. MTA carrier was used to place the materials into the canal. Following an orthograde method, a 4 mm thick apical plug of MTA and Biodentine was meticulously condensed using a hand plugger (GDC Fine Crafted Dental Pvt. Ltd, India) in groups G1 and G2. Radiographs were then captured for both G1 and G2 (Fig. 4) to verify the exact positioning of each apical plug. The dimensions of the plugs were meticulously measured using a digital ruler to ensure precise placement and uniformity in the experimental procedure. The teeth’ canal spaces were exclusively filled with thermoplasticized gutta-percha(Gutta-Smart, Dentsply Sirona, Advena Ltd, Malta). Radiographic evaluation was performed to observe the proper obturation in the G1, G2 & G3 groups, respectively (Fig. 5). Using a heat carrier, 2 mm of coronal gutta-percha was removed and vertically condensed. Subsequently, the coronal access was sealed using glass ionomer cement.

**Fig. 6:**
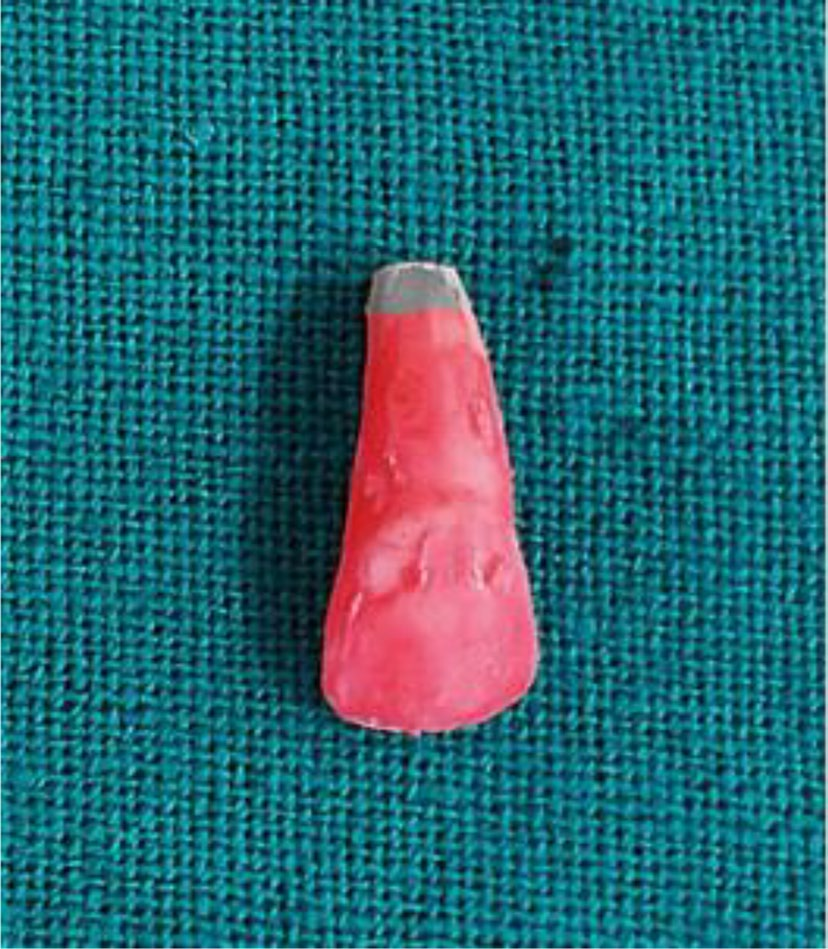
Application of nail varnish

**Fig. 7:**
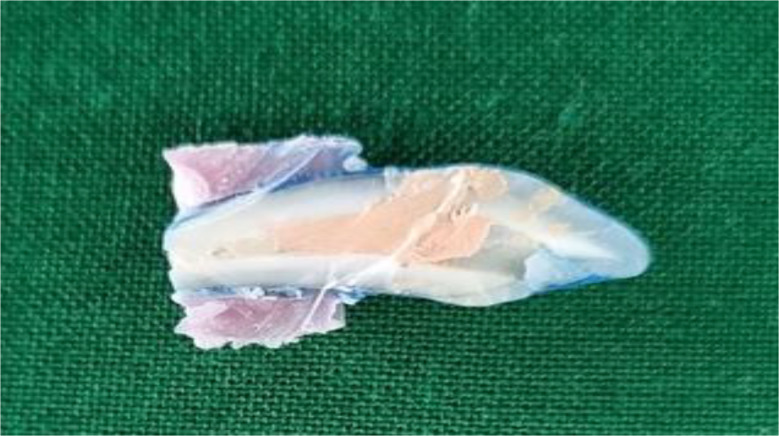
Longitudinal sectioning of specimen using diamond disc

Two coats of nail polish were applied to the external surface, excluding a 2 mm area from the apices (Fig. 6). All the specimens were immersed in a 1% methyl blue dye solution(Fisher Scientific, Lucknow) for 48 h, followed by a 5-minute wash under tap water. Acrylic blocks(DPI-RR Cold Cure, India)were crafted to mount each tooth. Diamond discs were employed to divide the specimens longitudinally (Fig. 7).

**Fig. 8:**
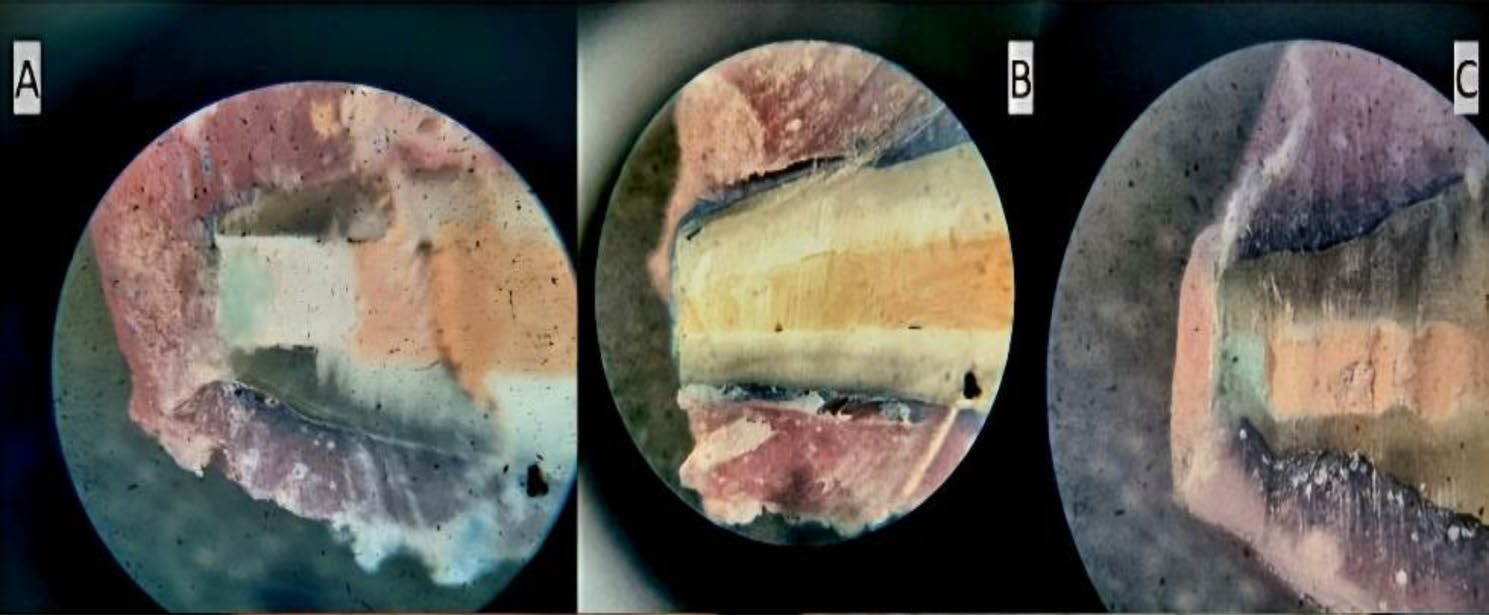
(**A**, **B** & **C**) Penetration of dye seen under stereomicroscope in G1, G2, G3 respectively

**Fig. 9:**
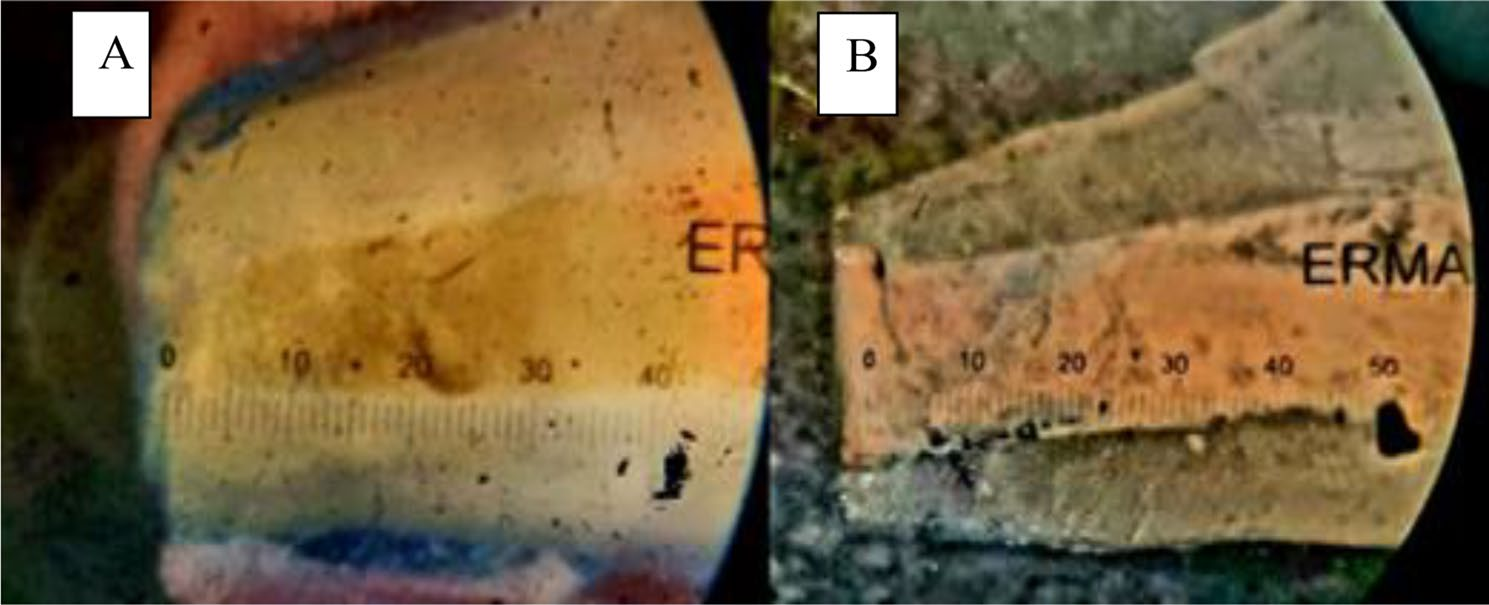
(**A** & **B**) Minimum and maximum penetration of dye measured in millimeters

One of each section was observed using a stereomicroscope at a magnification of 20x, equipped with a graded eyepiece(Erma, Japan). In the study, dye penetration measurements were conducted to assess the extent of leakage. Specifically, each sample’s most extended dye trace in millimetres was meticulously recorded across all three experimental groups. This method allowed for a comprehensive evaluation of the sealing efficacy and barrier function of the materials used in the study (Figures 8 and 9).

### Statistical analysis

SPSS (Statistical Package for Social Sciences) version 21. (IBM SPASS statistics [IBM corporation: NY, USA]) was used to perform the statistical analysis. Data was entered in the Excel spreadsheet, and descriptive statistics of the explanatory and outcome variables were calculated using the mean. Kruskal-Wallis test was applied to compare the mean apical microleakage among the groups with the post-hoc Mann-Whitney test for inter-group comparison. The level of significance was set at 5%.

## Results

Out of the initial 128 extracted teeth, 114 were chosen for the study and randomly divided into three groups: G1 (MTA), G2 (Biodentine), and G3 (Control), with 38 teeth in each group. After excluding 5 teeth from each group due to problems such as canal calcification, breakage during sectioning, and procedural errors, and to maintain equal distribution, a total of 33 teeth from each group were ultimately analyzed. Mean apical microleakage was higher in the Control group (1.62), followed by MTA (0.67)and Biodentine (0.16).

**Fig. 10:**
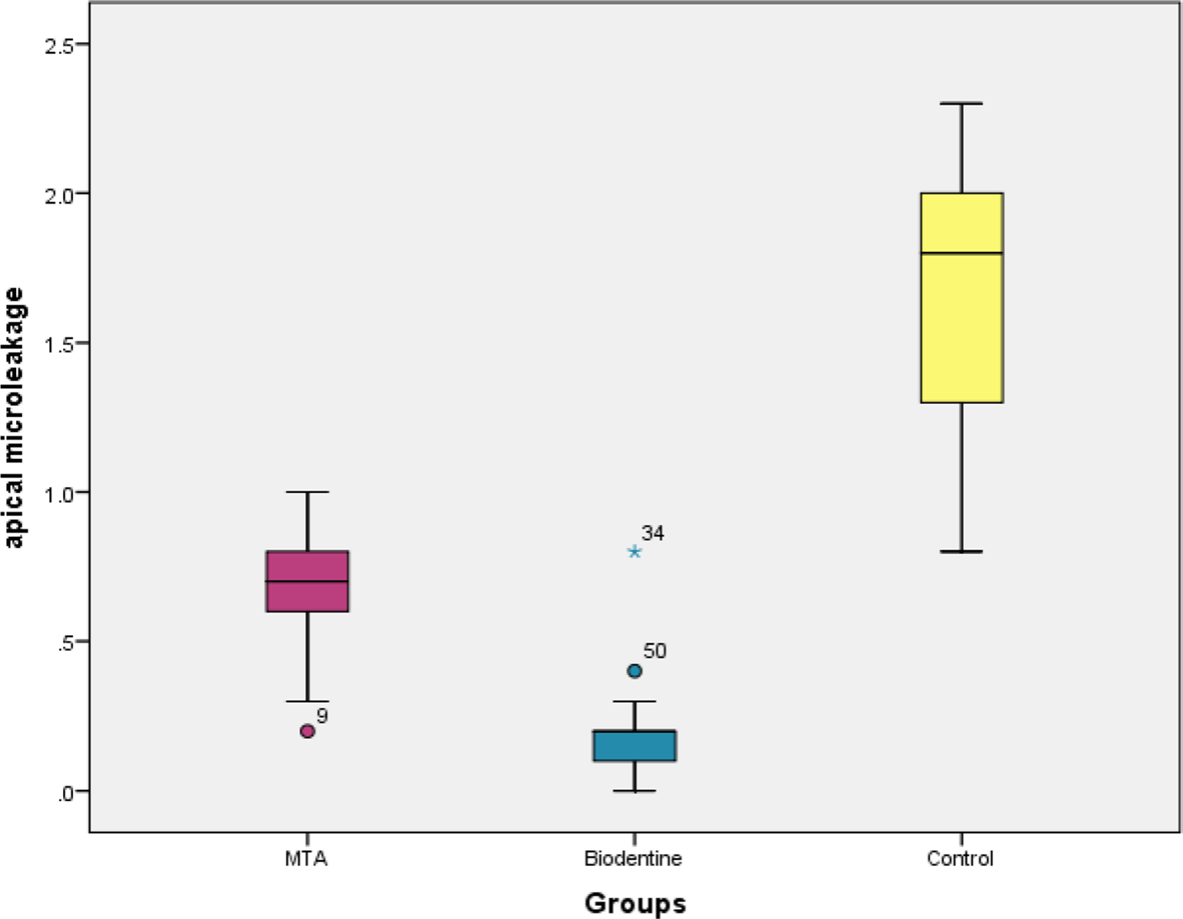
Graphical representation of inter-group comparison of the apical microleakage

The Kruskal-Wallis test was applied to compare the apical microleakage among the groups. Kruskal-Wallis test showed a statistically significant difference among the groups (*p = 0.001*) (Table 1). Inter-group comparison of apical microleakage between the groups was computed using a post-hoc Mann-Whitney test. A statistically significant difference was seen between all the groups: MTA Vs Biodentine, MTA Vs Control, and Biodentine Vs Control (*p = 0.001*) (Table 2) (Boxplot- Fig. 10).

**Table 1:** Comparison of the apical microleakage among the groups using Kruskal-Wallis

Groups	Minimum	Maximum	Mean	Standard Deviation	Variance	*p* value
MTA(G1)	0.2	1.0	0.67	0.207	0.042	0.001*
Biodentine(G2)	0.0	0.8	0.16	0.150	0.022	
Control(G3)	0.8	2.3	1.62	0.416	0.173	

^*^statistically significant

**Table 2:** Inter-group comparison of the apical microleakage using post-hoc bonferroni

Groups	U value	*p* value
MTA Vs Biodentine (G1 vs. G2)	40.00	0.0001*
MTA Vs Control(G1 vs. G3)	20.00	0.0001*
Biodentine Vs Control(G2 vs. G3)	0.50	0.0001*

^*^statistically significant

## Discussion

Necrotic teeth exhibiting immature apical foramina typically result from multiple factors affecting young permanent teeth, including trauma, caries and apical fractures [13, 14]. The aim of apexification is to establish an apical barrier to hinder the infiltration of toxins and bacteria from the root canal into periapical tissue. The barrier is essential to facilitate the compaction of root-filling material [15]. The study found that mean apical microleakage values were highest in the Control group (1.62), followed by MTA (0.67) and Biodentine(0.16). The significant difference in apical microleakage among the groups *(p = 0.001)*, indicate that MTA, Biodentine, and the Control group demonstrate distinct abilities to seal apical areas, highlighting their potential effectiveness as barriers in endodontic treatments.

After the introduction of MTA by Torbinejadet al.(1999), it emerged as the preferred material for apexification procedures [16]. The superiority of MTA plugs over traditional CaOH has often been emphasized, asserting that direct interaction with pulp and peri radicular tissues can prevent microleakage and promote tissue regeneration. Biodentine represents a novel bioactive dentine substitute cement. The liquid accompanying the cement powder mixing includes a water-soluble polymer and calcium chloride, which expedites the setting reactions [17].

However, despite the advancement, microleakage remains a significant cause of restoration failure. Microleakage refers to the infiltration of bacteria molecule ions between the margins of the tooth and restoration [18]. This study was designed as an in-vitro investigation to evaluate the apical microleakage of e-MTA and Biodentine when employed as orthograde apical plugs. The study also used the dye penetration method to compare their sealing abilities. While the dye penetration method has been widely used in microleakage studies, we acknowledge its limitations in accurately simulating clinical conditions. One significant concern is that the dye molecules may not adequately represent the behavior of bacteria or other clinical contaminants due to their smaller size. This limitation could potentially result in an overestimation of the sealing ability of the materials tested. Previous studies have suggested alternative methods, such as glucose leakage models, bacterial penetration tests, and fluid filtration techniques, which may provide a more accurate assessment of microleakage in clinical scenarios [19–21]. For example, a study [22] used a glucose leakage model and reported different outcomes compared to the dye penetration method, indicating that the latter might not fully capture the complexities of microleakage in vivo.

Maxillary anteriors were chosen due to their easy root canal morphology. CBCT assessments have shown that 97-99% of these teeth exhibit a single-rooted canal [23].

In the current study, the apical 2 mm of the root was resected; after that, a peeso reamer was used in an orthograde manner (1–6) followed by a retrograde approach (1–6) to simulate an open apex to approximate Cvek’s stage 3 root development. The periapical region was simulated for the first time using putty material in the present study. Putty is used in periapical simulations to replicate the soft tissue and bone structures around the apex of a tooth. Its moldable nature allows it to mimic the periapical region accurately. Magro et al.(2017) & Anamika et al. (2020) used lyophilized collagen sponge and platelet-rich fibrin membranes, respectively, as internal matrices [24, 25].

Kids e-MTA is a new endodontic material available in powder and liquid forms; e-MTA is touted to possess favourable handling characteristics, a fast-setting time, high compressive strength, and excellent resistance to washout. However, as of now, there is a lack of studies or literature substantiating these claims. In our research, we employed pre-dose capsulated formation for biodentine. This approach helped minimize fluctuations in the water/powder ratio, ensuring a consistent and homogenous mixture using an amalgamator. Additionally, these modifications have significantly improved the physical properties of Biodentine.

In our study, a 4 mm thick apical plug was made to ensure good apical sealing. Similar research was conducted by Abbas et al.(2020)and Bani et al.(2015), which showed that a 4-mm plug offered a superior seal, regardless of the material employed [26, 27]. Thermoplasticized gutta-percha is more malleable, ensuring better penetration into intricate canal irregularities and accessory canals than traditional methods. This improved adaptation minimizes gaps, reducing the risk of bacterial contamination and enhancing treatment success [28]. The current study assessed microleakage by immersing the solution in a 1% methylene-blue dye solution for 48 h. The 1% methylene-blue dye solution was chosen because it can penetrate farther than other dyes due to its more minor molecular size range of 0.5 to 0.7 nanometers [29]. Thapaliya et al.(2021) utilized the dye penetration method to assess microleakage, and Biodentine showed better sealing ability [10]. Cechellaet al.(2018) reported a contradictory result using a glucose leakage model; Biodentine demonstrated inferior sealing abilities compared to other materials [30]. Rafaei et al.(2020)concluded that Biodentine exhibited superior sealing efficiency than other materials [11]. Ozbay et al.(2014)stated that MTA displayed lower levels of microleakage than Biodentine [12]. Radevaet al.(2014) used the dye penetration method to evaluate the microleakage between MTA and Biodentine, but the difference was not statistically significant *(p > 0.05)* [31].

Biodentine achieves superior apical sealing relative to MTA due to its advanced handling properties, better flow characteristics, and superior adaptation to root canal walls. Furthermore, its chemical composition accelerates mineralization, contributing significantly to its enhanced sealing capabilities compared to MTA. These attributes make Biodentine a favoured option in clinical settings where reliable root canal sealing, and successful treatment outcomes are paramount.

### Limitations

- During the process of condensing the material to form the apical plug, certain samples exhibited extrusion beyond the apex.
- Given that this study is conducted in vitro, it may not fully replicate clinical conditions. Therefore, additional research is necessary to validate the relevance of these findings in practical clinical settings.

## Conclusion

The current study’s findings indicate that all three experimental groups showed evidence of microleakage, suggesting that some amount of fluid or dye penetrated through the root canal fillings in each group. The control group exhibited the highest level of microleakage, followed by the MTA and then the Biodentine group, with a decreasing trend in microleakage severity. These results suggest that Biodentine offers superior sealing efficiency compared to MTA when used as an artificial apical barrier in simulated young permanent teeth. Further, underscores Biodentine’s potential advantage in reducing the risk of microleakage and improving the overall success of endodontic treatments in clinical practice.

## Data Availability

The datasets used and analyzed during the current study are available from the corresponding author upon reasonable request.
